# Association of preoperative conization with recurrences after laparoscopic radical hysterectomy for FIGO 2018 stage IB1 cervical cancer

**DOI:** 10.1007/s00404-022-06816-6

**Published:** 2022-11-03

**Authors:** Yan Ding, Xuyin Zhang, Junjun Qiu, Chunbo Li, Keqin Hua

**Affiliations:** Department of Gynecology, Obstetrics and Gynecology Hospital of Fudan University, No. 128 Shenyang Road, Shanghai, 200090 China

**Keywords:** Cervical cancer, Laparoscopy, Radical hysterectomy, Conization, Recurrence

## Abstract

**Objective:**

To evaluate association of preoperative conization with recurrences after laparoscopic radical hysterectomy (LRH) for FIGO 2018 stage IB1 cervical cancer.

**Methods:**

This is a retrospective single-center study. Patients who underwent LRH for cervical cancer with squamous, adenosquamous and adenocarcinoma subtype from January 2014 to December 2018 were reviewed. All patients were restaged according to the 2018 FIGO staging system. Those who were in FIGO 2018 stage IB1 met the inclusion criteria. General characteristics and oncologic outcomes including recurrence-free survival (RFS) were analyzed.

**Results:**

A total of 1273 patients were included in the analysis. 616 (48.4%) patients underwent preoperative biopsy, and 657 (51.6%) patients underwent conization. Residual disease was observed in 822 (64.6%) patients. During a median follow-up of 50.30 months, 30 (2.4%) patients experienced recurrence. The univariate analysis showed that patients who had larger tumor diameter, the presence of residual tumor at final pathology, and underwent adjuvant treatment had a significant higher risk of recurrence (*P* < 0.01). Conversely, patients who underwent conization were significantly less likely to experience recurrence (*P* = 0.001). In the multivariate analysis, the independent risk factor associated with an increased risk of recurrence was resident macroscopic tumor (HR: 38.4, 95% CI 4.20–351.64, *P* = 0.001). On the contrary, preoperative conization was associated with a significantly lower risk of recurrence (HR: 0.26; 95% CI 0.10–0.63, *P* = 0.003). The Kaplan–Meier curves showed patients who underwent conization had improved survival over those who underwent biopsy (5 year RFS: 98.6 vs 95.1%, *P* = 0.001). The 5 year RFS of patients with residual tumor was significantly different (R0: 99.2%, R1: 97.4%, R2: 93.6%, *P* < 0.001), especially the patients with residual macroscopic tumor after conization (R0: 99.5%, R1: 99.0%, R2:92.4%, *P* = 0.006).

**Conclusion:**

Preoperative conization and the absence of residual tumor at the time of surgery might play a protective role in patients with FIGO 2018 IB1 cervical cancer following LRH, which support the theory of the influence of intraoperative tumor spread during radical hysterectomy. Further prospective evidence is needed.

## What does this study add to the clinical work

**Table:** 

Presence of residual tumor at time of surgery represented an independent predictor of recurrence for patients with cervical cancer stage IB1 following laparoscopic radical hysterectomy, while preoperative conization and absence of residual tumor might play a protective role. Further prospective evidence is needed.	

## Introduction

Cervical cancer is the fourth most common cancer in women worldwide. Surgery is the preferred treatment for women with early-stage cervical cancer. Over the last decades retrospective studies and prospective investigations have shown that minimally invasive surgery (MIS) was associated with less morbidity and similar survival outcomes than laparotomy [1, 2]. However, the recent results of the Laparoscopic Approach to Cervical Cancer (LACC) trial showed that minimally invasive radical hysterectomy was associated with lower rates of disease-free survival and overall survival (OS) than open radical hysterectomy for early-stage cervical cancer [3]. Similarly, increased recurrence and mortality rates with the MIS approach compared with laparotomy have been reported in a large retrospective cohort study [4]. The publication of these results triggered extensive discussion in clinical practice. Further retrospective or comparative analyses followed, some confirming the results of the LACC trial and others showing equivalent survival rates after MIS compared with open radical hysterectomy [5–9]. Contradictory results of various studies lead to the hypothesis that reduced survival after MIS might depend on failure to prevent tumor cell contamination through the use of uterine manipulators, intracorporal colpotomy or lack of vaginal cuff closure [10–13]. Recent studies showed that patients who underwent preoperative conization appear to have excellent survival even after laparoscopic surgery [14–19]. However, the enrolled population in these studies was based on the preoperative diagnosis with confounding factors such as larger tumor size, lymph node metastasis, and different surgical approach. In this retrospective study, patients were restaged the diagnosis from the 2009 International Federation of Gynecology and Obstetrics (FIGO) staging system to the new 2018 FIGO staging system, and those who diagnoses as FIGO 2018 stage IB1 cervical cancer were analyzed to explore the factors that influence the probability of recurrence after laparoscopic radical hysterectomy (LRH), especially the effect of preoperative conization.

## Material and methods

### Patients

This is a single-center retrospective analysis. The study was approved by the institutional review board of the Obstetrics and Gynecology Hospital of Fudan University, Shanghai, China (No. 2021-34). Patients who underwent LRH (Piver-Rutledge type II or type III) with sentinel lymph node mapping or retroperitoneal staging for cervical cancer from January 2014 to December 2018 in the Obstetrics and Gynecology Hospital of Fudan University were collected. All the procedures were accomplished with the use of a uterine manipulator and without vaginal closure and tumor exclusion before the colpotomy. Patients with squamous, adenocarcinoma and adenosquamous histologic subtypes at the final histologic evaluation were all reassessed and restaged according to the new 2018 FIGO staging system. Those who diagnosed as FIGO 2018 stage IB1 were finally analyzed. Exclusion criteria were: (1) preoperative chemotherapy or radiotherapy, and (2) pre-existing or concurrent malignancies.

The surgical procedures were performed by 36 certified gynecologic oncologists. The patients were stratified by the type of preoperative diagnosis performed: cervical biopsy or cervical conization (either cold knife cone or loop electrosurgical excision procedure). According to the size of the residual tumor in the final pathology, patients were divided into three subgroups: R0 (no residual lesion), R1 (residual microscopic visible tumor), R2 (residual macroscopic visible tumor). After surgery, patients underwent adjuvant therapy if they presented any intermediate-risk factors met the Sedlis criteria [20] or the “four-factor model” [21]. According to the National Comprehensive Cancer Network (NCCN) guidelines, patients were followed up every 3 months for 2 years, every 6 months for the next 3 years, and once per year thereafter.

### Data collection

Case records were retrieved from the hospital information system and the outpatient information system which contained information on age, clinical diagnosis, surgical treatment, pathology reports, and postoperative adjuvant treatment. Survival data were abstracted from the follow-up information system which was updated on a regular basis. The last follow-up date of documented event was January 2021. The occurrence of recurrence and death within the follow-up period was registered. Recurrences were classified based on number and location.

### Statistical analyses

Statistical analyses were performed with SPSS v23.0 (IBM Corp., Armonk, NY). Student’s *t* test or analysis of variance was used to compare continuous variables, whereas chi-square test was used to compare categorical variable*.* Recurrence-free survival (RFS) was defined as the length of time from the primary surgery to initial diagnosis of recurrence or date of last follow-up. Time to recurrence was calculated as the time difference in months between surgery and first evidence of recurrent disease. OS was calculated as the difference between the primary surgery date and the date of death from cervical cancer or last contact, whichever came first. The Kaplan–Meier curves were used to perform univariate survival analyses. The log-rank test was conducted for significance analysis. The associations of variables with RFS were evaluated using Cox proportional hazards regression models with backward Wald. Hazard ratios (HR) were presented with 95% confidence intervals (CI). Differences were considered to be statistically significant at *P* < 0.05.

## Results

**Fig. 1:**
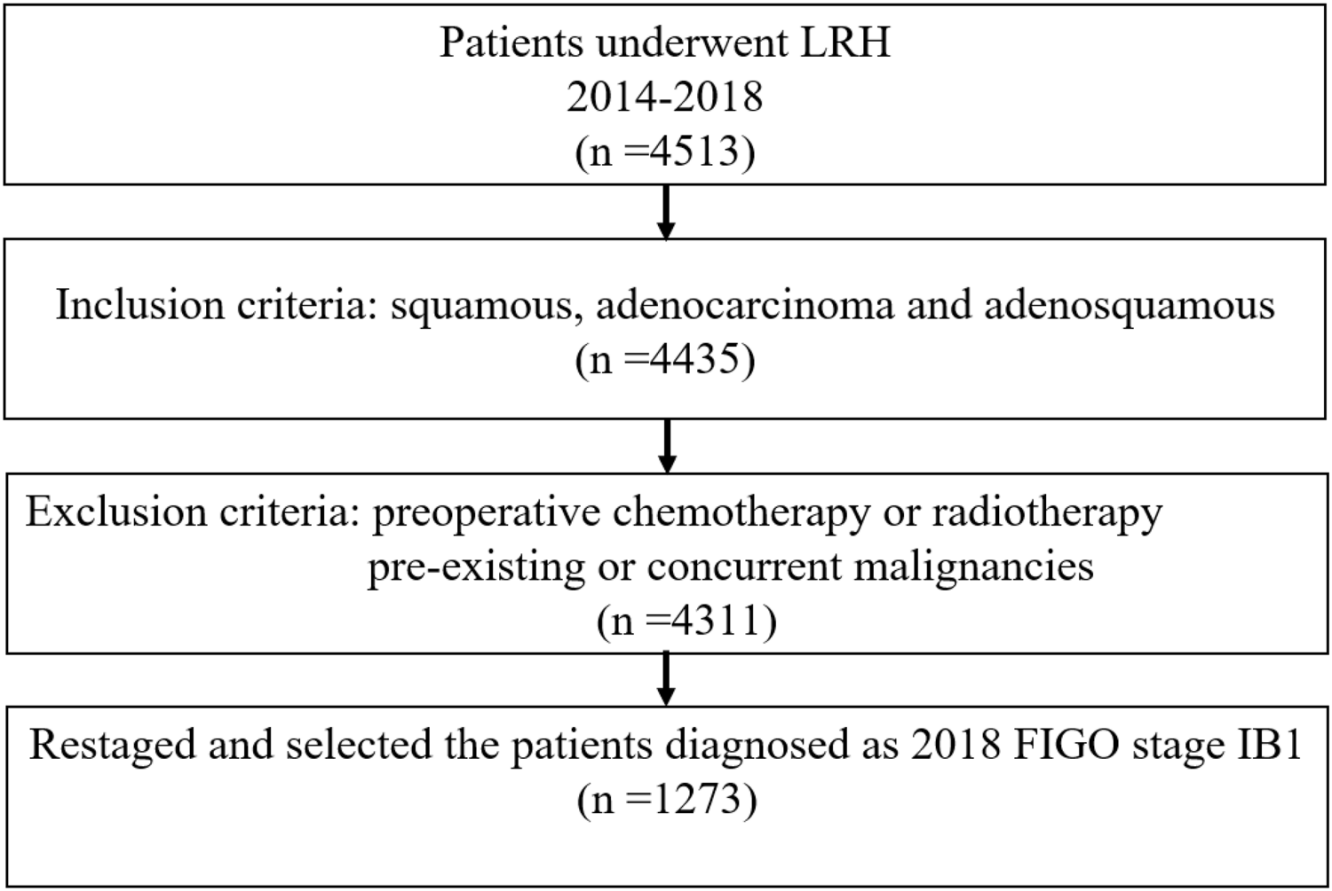
The flow chart of study design

Over the study period, a total of 4688 patients with cervical cancer underwent radical hysterectomy, and 1273 patients with stage IB1 according to the new FIGO 2018 staging system met the criteria eventually (Fig. 1). As seen in Table 1, the average age of patients was 47.00 ± 9.21 years. Most patients (74.1%) were squamous cell carcinoma. The median tumor diameter was 10.51 ± 0.50 mm. 877 cases (68.9%) had tumor diameter smaller than 10 mm. 616 (48.4%) patients underwent cervical biopsy for diagnosis, and 657 (51.6%) patients underwent preoperative cervical conization. Residual disease was observed in 822 (64.6%) patients, with 367 (28.8%) residual macroscopic carcinoma. Mean 22.7 days after diagnosis, patients underwent surgical treatment. According to the NCCN guidelines, 165 (13.0%) patients with intermediate risk required adjuvant therapy, but 4.4% patients did not complete adjuvant therapy.

**Table 1:** Characteristics of the patient with 2018 FIGO stage IB1 cervical cancer

	Total (*n* = 1273)
Age (years)	47.00 ± 9.21
Conization	
No	616 (48.4)
Yes	657 (51.6)
Histological type	
SCC	943 (74.1)
AC	248 (19.5)
ASC	82 (6.4)
Pathology tumor size (mm)	10.51 ± 0.50
6–10	877 (68.9)
11–15	165 (13.0)
16–20	231 (18.1)
Stromal infiltration	
< 1/3	1085 (85.2)
[1/3–2/3)	49 (3.8)
≥ 2/3	139 (10.9)
LVSI	
No	1047 (82.2)
Yes	226 (17.8)
Tumor in the final specimen	
R0	451 (35.4)
R1	455 (35.7)
R2	367 (28.8)
Adjuvant treatment	
Unrequired	1108 (87.0)
Completed	109 (8.6)
Uncompleted	56 (4.4)
Time to operation (days)	22.72 ± 13.76
Recurrence	30 (2.4)
Time to recurrence (months)	26.40 ± 17.28
Site of recurrence	
Vaginal recurrence	1 (3.4)
Pelvic recurrence	17 (58.6)
Abdominal recurrence	11 (37.9)
Distant recurrence	13 (43.3)
Distribution of recurrence	
Single site	18 (60.0)
Multiple site	12 (40.0)
Death of disease	16 (1.3)

*LVSI* lymphovascular space incision; *SCC* squamous cell carcinoma; *AC* adenocarcinoma; *ASC* adenosquamous carcinoma; *R0* no residual disease; *R1* residual microscopic tumor; *R2* residual macroscopic tumor

During a median follow-up of 50.30 months (2–84 months), 30 (2.4%) patients experienced recurrence, and 16 (1.3%) died of disease. The median time to recurrence was 31.3 months (2–60 months). Most patients (60.0%) had a single site recurrence. Seventeen (58.6%) patients recurred in the pelvic, 11 (37.9%) experienced abdominal recurrence, and 13 (43.3%) recurred distantly.

The univariate analysis of factors associated with recurrence showed that patients who had larger tumor diameter (HR: 3.39, 95%CI 1.78–6.48, *P* < 0.001), the presence of residual tumor at final pathology (HR: 2.40, 95%CI 1.44–3.98, *P* = 0.001), and underwent adjuvant treatment (HR: 2.03, 95%CI 1.26–3.25, *P* = 0.003) had a significant higher risk of recurrence. Conversely, patients who underwent preoperative conization were significantly less likely to experience recurrence (HR: 0.24, 95%CI 0.10–0.57, *P* = 0.001) (Table 2). In the multivariate analysis, the independent risk factor associated with an increased risk of recurrence was residual tumor at final histology. Patients with resident macroscopic tumor had a 38.41 (95% CI 4.20–351.64, *P* = 0.001) increased risk for recurrent disease. On the contrary, preoperative conization was associated with a significantly lower risk of recurrence (HR: 0.26; 95% CI 0.10–0.63, *P* = 0.003) (Table 3).

**Table 2:** Univariate regression analysis on recurrences following laparoscopic radical hysterectomy for 2018 FIGO stage IB1 cervical cancer

		HR	95%CI	*P*
Age (years)		1.03	0.99–1.07	0.141
Histological type				
	SCC	1		0.153
	AC	1.71	0.75–3.94	0.205
	ASC	2.60	0.88–7.68	0.084
Tumor size (mm)		3.39	1.78–6.48	< 0.001
	6–10	1		0.003
	11–15	1.14	0.33–3.97	0.836
	16–20	3.61	1.70–7.69	0.001
Conization				
	No	1		
	Yes	0.24	0.10–0.57	0.001
Tumor in the final specimen		2.40	1.44–3.98	0.001
	R0	1		0.003
	R1	3.29	0.91–11.96	0.070
	R2	7.27	2.13–24.83	0.002
Stromal infiltration				
	< 1/3	1		0.925
	[1/3–2/3)			
	≥ 2/3	1.24	0.43–3.54	0.694
LVSI				
	No	1		
	Yes	2.06	0.94–4.50	0.070
Adjuvant treatment		2.03	1.26–3.25	0.003
	Unrequired	1		0.013
	Completed	1.97	0.68–5.75	0.213
	Uncompleted	4.13	1.55–11.01	0.005

*LVSI* lymphovascular space incision; *SCC* squamous cell carcinoma; *AC* adenocarcinoma; *ASC* adenosquamous carcinoma; *R0* no residual disease; *R1* residual microscopic tumor; *R2* residual macroscopic tumor

**Table 3:** Multivariate regression analysis on probability of recurrences following laparoscopic radical hysterectomy for 2018 FIGO stage IB1 cervical cancer

		HR	95%CI	*P*
Pathology tumor size (mm)		2.54	0.40–15.81	0.323
	6–10	1		0.183
	11–15	1.01	0.11–1.41	0.155
	16–20	2.05	0.40–10.51	0.391
Conization				
	No	1		
	Yes	0.26	0.10–0.63	0.003
Tumor in the final specimen				
	R0	1		0.005
	R1	3.46	0.95–12.58	0.059
	R2	38.41	4.20–351.64	0.001
Adjuvant treatment				
	Unrequired	1		0.192
	Completed	1.17	0.39–3.54	0.778
	Uncompleted	2.59	0.93–7.23	0.069

*R0* no residual disease; *R1* residual microscopic tumor; *R2* residual macroscopic tumor

**Fig. 2:**
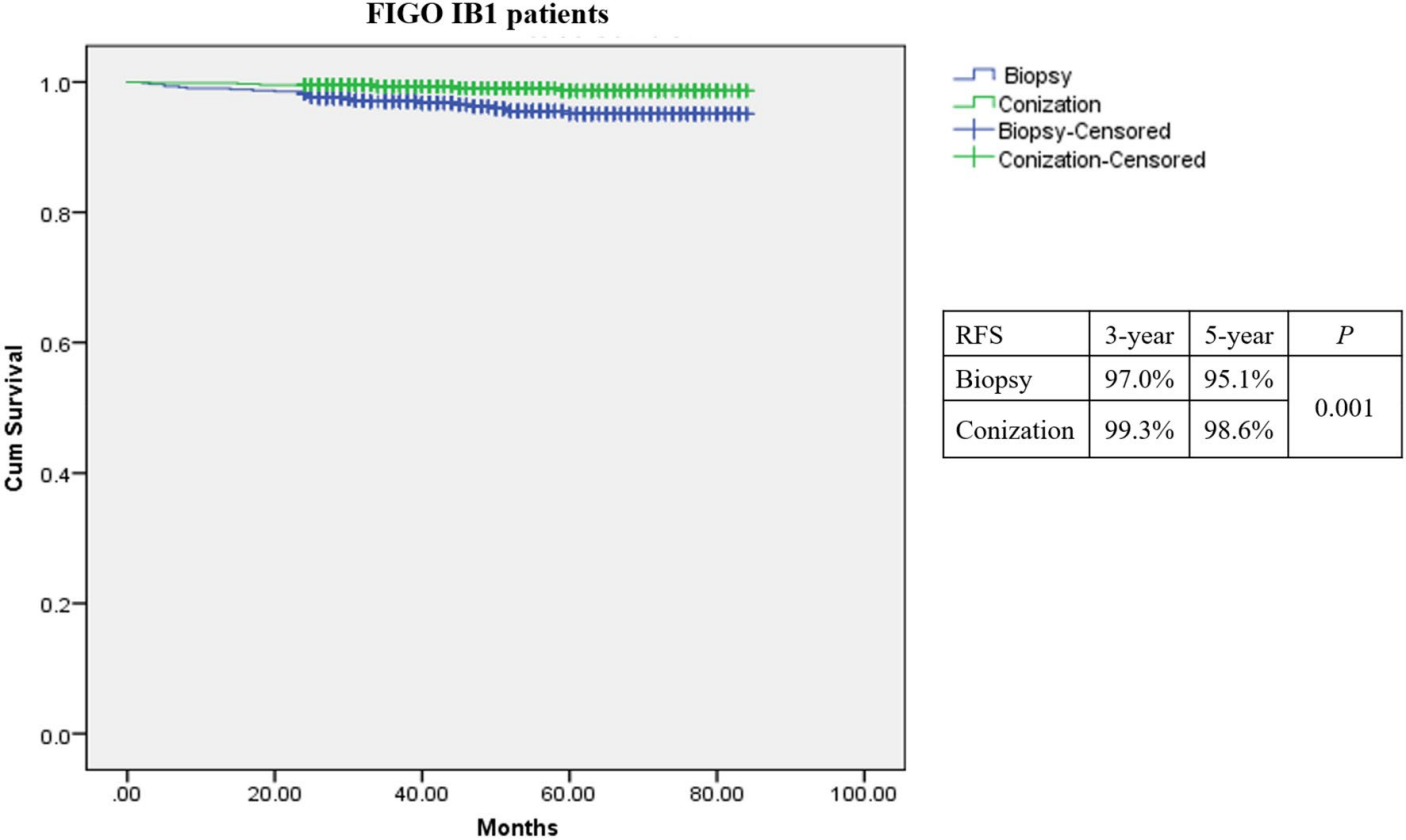
The Kaplan–Meier survival curves on recurrence-free survival (RFS) analysis of preoperative conization for FIGO (2018) stage IB1 patients

**Fig. 3:**
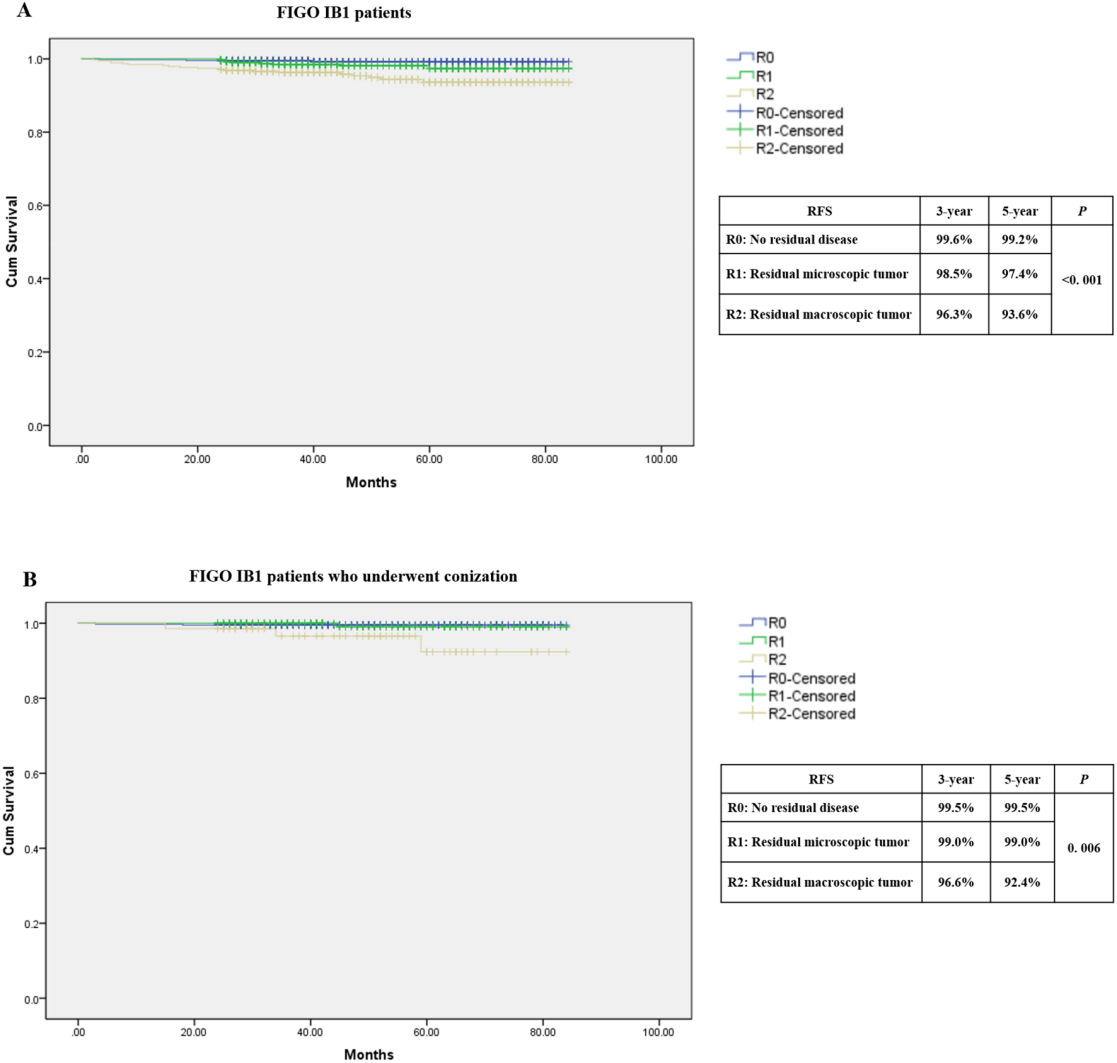
The Kaplan–Meier survival curves on recurrence-free survival (RFS) analysis of residual carcinoma in the final specimen for all of the FIGO (2018) stage IB1 patients (**A**) and patients who underwent conization (**B**)

The Kaplan–Meier curves showed patients who underwent conization had improved survival over those underwent biopsy. As seen in Fig. 2, the 5 year RFS of patients was 95.1% in biopsy and 98.6% in conization (*P* = 0.001). The 5 year OS of patients was 97.2% in biopsy and 99.2% in conization (*P* = 0.035). As shown in Fig. 3A, the 5 year RFS of patients with residual tumor in the final specimen was significant difference (R0: 99.2%, R1: 97.4%, R2: 93.6%, *P* < 0.001). Patients who underwent conization were further analyzed to explore the effect of residual tumor on recurrence. It was found that there was significant difference in the recurrence rate of residual tumor at the final pathology after preoperative conization. As shown in the Fig. 3B, the 5 year RFS of patients was 99.5% in R0, 99.0% in R1, and 92.4% in R2, respectively (*P* = 0.006).

## Discussion

In the present study, residual macroscopic tumor in the final pathology was found to be the independent predictors of laparoscopic radical hysterectomy in patients with cervical cancer FIGO 2018 stage IB1. On the contrary, preoperative conization was the only protective factor associated with reduced risk for recurrence. Patients who underwent preoperative conization with macroscopic tumor removal were less likely to experience recurrence. These data support the theory of the influence of intraoperative tumor spread during radical hysterectomy.

After publication of the LACC trial, many studies have been published to discuss the possible reasons for the poor survival with the use of MIS compared to open radical hysterectomy. Aspects such as learning curves, the patient selection, standardization of surgical technique, pathology processing and reporting have been indicated as potential causes of the unexpected results of the LACC study; however, the manipulation of the tumor during surgery which resulted in tumor cell spread may represent the most reliable hypothesis associated with the unfavorable oncological outcomes of minimally invasive radical hysterectomy [10, 12, 15, 22–25]. One study mechanistically demonstrated that tumor cell spread may occur during intracorporal colpotomy when intravaginal tumor components contacted with the intraperitoneal cavity [11]. While all the surgical procedures were accomplished with the use of a uterine manipulator and without vaginal closure and tumor exclusion before the colpotomy in this study, which means tumor cell would be manipulated during surgery or exposed to the intraperitoneal cavity. We found that preoperative conization reduced the risk of recurrence by 64% in the population; on the other hand, patients with residual macroscopic tumor during surgery had a 38.41 increased risk of recurrence compared to those with no residual tumor in the cervix. These results were similar to those of recent studies. In the study of Casarin et al. [15] showed that preoperative conization reduced the risk of recurrence by 68%, while presence of residual tumor at final pathology increased risk of recurrence with an odds ratio (OR) 5.29 following laparoscopic surgery for early-stage cervical cancer. Klapdor et al. [18] found that in multivariate analysis preoperative conization was the only factor significantly associated with reduced risk for recurrences with an OR 5.90. Uppal et al. [26] reported that conization before minimally invasive radical hysterectomy was associated with lower recurrence risk by 60%.

The results of these studies indicate that preoperative conization plays a potentially protective role in patients with early-stage cervical cancer. Meanwhile the results also raise another question whether resection of all macroscopic visible tumor reduces the chances for tumor cell spillage during colpotomy. Further analysis in our study showed that there was significant difference in the 5 year RFS among the presence of residual tumor. Patients with absence of residual tumor in the final specimen had significantly better RFS, while patients with residual macroscopic visible tumor had the worst RFS. Moreover, the similar results were found in patients who underwent preoperative conization. This suggested that patients having conization with tumor removal before surgery could be associated with improved outcomes, even presence of residual microscopic tumor at the final pathology. These results might support the thesis that preoperative removal of tumors by conization might overcome possible tumor spread occurring during the colpotomy at the time of laparoscopic radical hysterectomy [14]. The absence of residual disease in the cervix at the time of surgery might nullify the effect of the role of tumor manipulation. The results of some retrospective multicenter studies showed that LRH with enclosed colpotomy and without the use of uterine manipulator would have similar or even better survivals than open surgery [10, 13].

The previous studies showed that tumor size > 2 cm was the only factor that characterized patients with increased risk of recurrence, while patients having tumor size < 2 cm might benefit from the advantages of laparoscopic surgery [20, 27, 28]. In the present study, we focused on the patients whose tumor size was < 2 cm and divided the tumor size into three subgroups, which could better analyze the impact of tumor size on recurrence. It was found that the tumor size was associated with the recurrence but not the independent predictor of recurrence after LRH. This indicated that it is the residual tumor but not the initial tumor size that actually affect the recurrence of patients who underwent LRH, which also further support the thesis that the absence of residual disease in the cervix at the time of surgery might nullify the effect of the role of tumor manipulation for patients undergoing LRH. In future studies, the role of preoperative conization to reduce the visible tumor mass should be evaluated especially in laparoscopic surgery.

On the other hand, these results in the study may also give some indications for patients who desired to preserve their fertility. Absence of residual disease in the cervix was of great importance. There were some clinical trials recently which showed that cervical conization with lymph node evaluation may be a feasible conservation management in low-risk cervical cancer [29, 30]. Negative surgical margin (> 3 mm) after conization could be oncologic safe even in fertility-sparing patients with stage IB1 (≤ 2 cm). More prospective multi-institutional studies evaluating the efficacy and safety of conization for fertility-sparing patients in early-stage cervical cancer should be conducted.

Concerning the site of recurrence, the present study showed that patients who underwent LRH were more likely to develop intrapelvic and peritoneal recurrences. This is accordance with what reported by recent retrospective studies, which showed that patients undergoing laparoscopic radical hysterectomy are at higher risk of developing intrapelvic recurrences and peritoneal carcinomatosis compared to open surgery [15, 31]. Further evidence is needed.

This large sample of study was designed based on the final pathological staging which could exclude the high-risk factors such as positive pelvic nodes and positive surgical margin, but focus on the impact of cervical lesion and preoperative conization on recurrence. Moreover, all surgeries were performed by qualified surgeons in the high-volume hospital using standardized surgical technique which allows for a more reliable evaluation on the influence of patients and tumor characteristics compared to multicenter studies. Other strengths of our study include the large sample size, and standard reports of the pathologic review in our hospital which is particularly important if data on pathologic variables are analyzed. However, some limitations have to be mentioned. First, due to the retrospective study design, the results might suffer of few important intrinsic biases. Second, some patients received adjuvant chemotherapy or radiotherapy after surgery. But not all patients received adjuvant therapy in the same institution, so the effect of variation in irradiation technique and chemotherapeutic regimens cannot be eliminated. Third, we did not collect the data of patients treated with the open approach in the study period. Fourth, our data only reflected a single-center experience. As the study population was from a specific geographical area, further investigation at multiple centers is needed.

## Conclusion

In conclusion, the results of this large sample study showed that the presence of residual tumor at time of surgery represented an independent predictor of recurrence for patients with cervical cancer stage IB1 following laparoscopic radical hysterectomy. On the contrary, preoperative conization and the absence of residual tumor at the time of surgery might play a protective role. Our data support the theory of the influence of intraoperative tumor spread during radical hysterectomy. Analyses on larger series are needed to explore better the effect of preoperative conization in patients with cervical cancer. Further prospective trials are warranted to strengthen our findings.
